# Stability of oral and fecal microbiome at room temperature: impact on diversity

**DOI:** 10.3389/frmbi.2025.1334775

**Published:** 2025-04-30

**Authors:** Blanca Rius-Sansalvador, David Bars-Cortina, Olfat Khannous-Lleiffe, Ainhoa Garcia-Serrano, Elisabet Guinó, Ester Saus, Toni Gabaldón, Victor Moreno, Mireia Obón-Santacana

**Affiliations:** ^1^ Unit of Biomarkers and Susceptibility (UBS), Oncology Data Analytics Program (ODAP), Catalan Institute of Oncology (ICO), L’Hospitalet del Llobregat, Barcelona, Spain; ^2^ ONCOBELL Program, Bellvitge Biomedical Research Institute (IDIBELL), L’Hospitalet de Llobregat, Barcelona, Spain; ^3^ Doctoral Programme in Biomedicine, University of Barcelona (UB), Barcelona, Spain; ^4^ Barcelona Supercomputing Centre (BSC-Centro Nacional de Supercomputación (CNS)), Barcelona, Spain; ^5^ Institute for Research in Biomedicine (IRB Barcelona), The Barcelona Institute of Science and Technology, Barcelona, Spain; ^6^ Consortium for Biomedical Research in Epidemiology and Public Health (CIBERESP), Madrid, Spain; ^7^ Catalan Institution for Research and Advanced Studies (ICREA), Barcelona, Spain; ^8^ Centro de Investigación Biomédica En Red de Enfermedades Infecciosas (CIBERINFEC), Barcelona, Spain; ^9^ Department of Clinical Sciences, Faculty of Medicine and Health Sciences and Universitat de Barcelona Institute of Complex Systems (UBICS), University of Barcelona (UB), L’Hospitalet de Llobregat, Barcelona, Spain

**Keywords:** oral microbiome, fecal microbiome, stability study, GCAT, 16S, pilot study, room temperature

## Abstract

**Introduction:**

When collecting oral and fecal samples for large epidemiological microbiome studies, optimal storage conditions such as immediate freezing are not always feasible. It is essential to study the impact of temporary room temperature (RT) storage on microbiome diversity.

**Methods:**

We conducted a pilot study to validate a sampling protocol based on the viability of 16S rRNA gene sequencing in microbiome samples. Fecal and oral samples from five participants were collected and preserved under different conditions: a) 70% ethanol; b) FIT tube for stool; and c) chlorhexidine solution for oral wash. Four aliquots per sample were stored at RT and frozen at days 0, 5, 10, and 15.

**Results:**

Alpha diversity showed a maximum average decrease of 0.3%, 1.6%, and 1.7% after 5 days for oral, stool in ethanol, and stool in FIT samples, respectively. The relative abundances of the main phyla and orders remained stable throughout the 15 days.

**Discussion:**

Microbiome diversity appears remarkably resilient. Fecal and oral samples stored at RT in 70% ethanol, chlorhexidine, and FIT tubes exhibited minimal changes over 15 days. These results support the feasibility of large-scale microbiome studies with delayed sample processing.

## 1 Introduction

Gut and oral dysbiosis has been associated with the development and progression of some diseases in recent years. For instance, a role of the microbiota has been suggested in an enormous variety of diseases (Bull and Plummer, 2014; Durack and Lynch, 2019; Lu et al., 2019; Willis and Gabaldón, 2020; Chen et al., 2021; Fan and Pedersen, 2021; Martínez et al., 2022; Tuganbaev et al., 2022) including metabolic disorders, systemic, cardiovascular, liver, psychological or mental, and neurodegenerative diseases, arthritis, and cancer, such as gastrointestinal, among others. As many aspects of the relationship between the microbiome and diverse diseases are still unknown (Malla et al., 2019), the study of microbiota is an emerging field that is enhancing its knowledge. When collecting samples for microbiome analysis, several procedures and methodologies are used, hindering comparisons across studies. Immediate freezing has been considered the best practice for microbiome preservation (Song et al., 2016; Ilett et al., 2019; Moossavi et al., 2019); however, this approach is not feasible for large epidemiological studies that aim to obtain samples shipped by postal mail. In these cases, the samples use to remain for a few days at room temperature until they arrive at their destination (Williams et al., 2019; Young et al., 2021; Soriano et al., 2022).

Previous research has studied the stability of fecal and oral 16S rRNA gene sequencing microbiome samples (Dominianni et al., 2014; Choo et al., 2015; Flores et al., 2015; Sinha et al., 2016; Song et al., 2016; Gudra et al., 2017; Byrd et al., 2019; Bescos et al., 2020; Park et al., 2020; Krigul et al., 2021; Marotz et al., 2021). Regarding fecal microbiome collection methods, 70%–99% ethanol has historically been the most popular stabilization media (Park et al., 2020). However, there are fewer studies about the storage of the samples at room temperature compared to other collection methods, such as the Flinders Technology Associates (FTA) or the Fecal Occult Blood Test (FOBT) (Byrd et al., 2019).

Furthermore, a widely used collection technique for cancer screening is the Fecal Immunochemical Test (FIT). Some metagenomic studies recommend the use of FIT in cohort studies since the microbial profile stability of the samples stored for one week at room temperature has been validated (Gudra et al., 2017; Byrd et al., 2019; Krigul et al., 2021).

The room temperature stability of other fecal microbiome collection methods has been proven for FTA cards at 8 weeks (Song et al., 2016), OMNIgene Gut Kit for 3 days (Choo et al., 2015) and 8 weeks (Song et al., 2016; Park et al., 2020), FOBT for 3, 4 and 7 days (Dominianni et al., 2014; Sinha et al., 2016; Gudra et al., 2017; Byrd et al., 2019; Wu et al., 2021), RNAlater for 3, 4 and 7 days (Choo et al., 2015; Flores et al., 2015; Sinha et al., 2016; Byrd et al., 2019; Wu et al., 2021) and 8 weeks (Park et al., 2020).

Regarding the oral microbiome, previous studies used Scope^®^ oral wash (mainly composed of Alcohol, Domiphen Bromide and Cetylpyridinium Chloride) to preserve oral microbiome samples (Vogtmann et al., 2019; Yano et al., 2020), as it has been demonstrated that samples preserved with Scope are stable in terms of alpha and beta diversity up to 4 days at RT (Vogtmann et al., 2019; Wu et al., 2021). However, as this solution is not easily found in Europe, Chlorhexidine oral wash (Lacer^®^) was used. Chlorhexidine has been commonly used in many clinical trials where effective results have been proven in reducing the proliferation of bacterial species (Eick et al., 2011; James et al., 2017; Brookes et al., 2020; Xiang et al., 2021). Furthermore, the effect of daily use of chlorhexidine oral wash on the oral microbiome has been studied, showing significant differences in the abundance of some phyla (Bescos et al., 2020) and a decrease in terms of alpha diversity compared with sputum samples (Chatzigiannidou et al., 2020; Pragman et al., 2020). Despite demonstrating that oral washes containing chlorhexidine are related to a major shift in the oral microbiome, the stability of the samples for microbiome analyses, when preserved at RT has not already been studied.

The long-term prospective cohort study of the Genomes for Life (GCAT) aims to facilitate the prediction and treatment of frequent chronic diseases as well as gauge the role of epidemiological, genomic and epigenomic factors (Obón-Santacana et al., 2018). As part of this project, oral and fecal samples for microbiome studies need to be collected across the Catalan territory (northeast Spain). Given the logistical challenge of postal sample transport, where storage duration at room temperature (RT) may vary, validating the sampling protocol is essential to ensure sample viability. This study focuses on evaluating the short-term stability of fecal samples (collected in 70% ethanol and FIT tubes) and oral samples (collected via 0.12% chlorhexidine oral wash) by analyzing changes in alpha and beta diversity and the distribution of major bacterial genera. Ensuring the microbial diversity remains stable for several days at RT is key to maintaining data quality for future large-scale collection.

## 2 Materials and methods

### 2.1 Sample collection

In this study, 5 volunteer individuals (3 women and 2 men, median age 37) provided three different types of samples for microbiome analysis: one oral wash, preserved in 0.12% chlorhexidine and two fecal samples, one preserved in a FIT tube (FIT, OCSensor, Eiken Chemical Co., Tokyo, Japan) and another in a 5 ml tube with 1 ml of 70% ethanol. Samples were collected at home. Participants were instructed to obtain oral samples in the morning, before any food or tooth brushing, by doing an oral rinse for 1 minute with Lacer^®^ oral wash and then spitting the content in a tube. Stool samples, if obtained the night before, were kept at 4°C before transport to the lab.

Upon arrival at the laboratory, the samples underwent the following processing steps. The oral rinse was transferred to a 15 ml tube and centrifuged at maximum speed, as allowed by the rotor of the centrifuge. The supernatant was discarded, and 1–2 ml of PBS 1X was added. The pellet was resuspended, transferred to a 2 ml Sarstedt tube, and centrifuged again at 2000–3000 rpm for 5 minutes. The s supernatant was discarded. The content of the FIT was transferred to a 2ml Sarstedt tube for further processing. The stool samples were thoroughly homogenized using a spatula or by inversion to ensure uniform consistency. Next, two 2ml aliquots were carefully prepared using a Pasteur pipette or a syringe. The aliquots were transferred into 2 ml Sarstedt tubes for further analysis.

**Figure 1:**
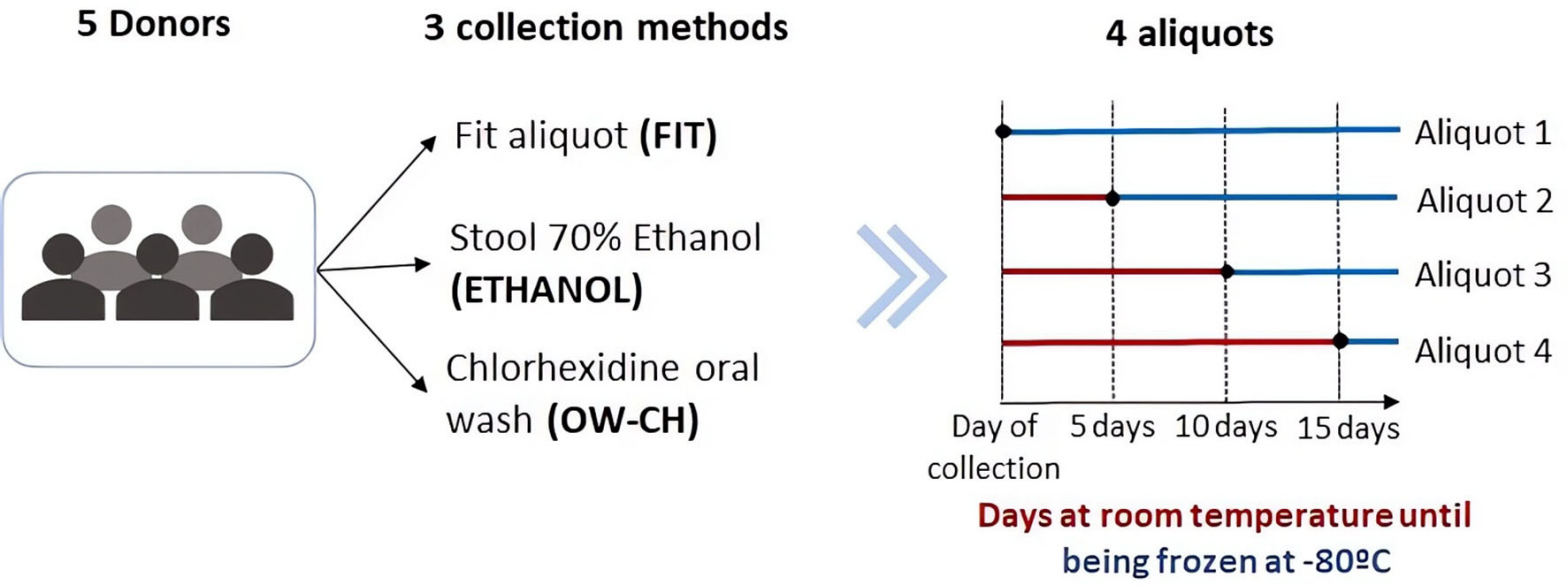
Schematic representation of the sample collection process.

For the three collection methods, a total of 4 aliquots of each sample were prepared and one aliquot was immediately frozen at −80°C until processing. The rest were consecutively frozen after remaining at room temperature for 5, 10 and 15 days, resulting in a total of 60 samples from 5 individuals at 4 time points (**Figure 1**; **Supplementary Table S1**). None of the participants took oral antibiotics, injected antibiotics, stomach protectors, or acid-lowering medication in the last 3 months. All individuals agreed to participate in the study and provided written informed consent. The University Hospital of Bellvitge ethics committee approved the protocol of the study (PR084/16).

### 2.2 DNA extraction and sequencing

DNA was extracted using the DNeasy PowerLyzer PowerSoil Kit (Qiagen, ref. QIA12855) following the manufacturer’s instructions with slight modifications depending on the initial sample type (FIT, oral wash and stool samples). Two negative controls of the DNA extraction process (with no initial sample) were also included. Briefly, for FIT samples, a pre-enrichment step was added by centrifuging the samples at 20,000 g for 5 min at 4°C. The supernatant was removed, and the pellet was resuspended in 750 μl of PowerBead Solution, mixed and transferred to a Bead tube with beads. Stool samples were already frozen in 2 ml tubes, where 750 μl of PowerBead Solution and the beads were directly added. For oral wash samples, pellets were resuspended in 750 μl of PowerBead Solution, mixed and transferred to a Bead tube with beads. From here on all samples were processed in the same way: 60 μl of Solution C1 was added, and samples were vortexed briefly and incubated at 70°C with shaking (700 rpm) for 10 min. The extraction tubes were then agitated in a horizontal vortex (Genie2) for 20 min at maximum speed. Tubes were centrifuged at 10,000 g for 3 min and the supernatant was transferred to a clean tube. Then, 250 μl of Solution C2 was added, and the samples were vortexed for 5 s and incubated on ice for 5 min. After 1 min of centrifugation at 10,000 g, 600 μl of the supernatant was transferred to a clean tube, 200 μl of Solution C3 was added, and the samples were vortexed for 5 s and incubated on ice for 5 min again. A total of 750 μl of the supernatant was transferred into a clean tube after 1 min centrifugation at 10,000 g. Then, 1,200 μl of Solution C4 was added to the supernatant, samples were blended by pipetting up and down, and 675 μl was loaded onto a spin column and centrifuged at 10,000 g for 1 min, discarding the flow through. This step was repeated three times until all samples had passed through the column. 500 μl of Solution C5 was added onto the column, and the samples were centrifuged at 10,000 g for 1 min. The flow through was discarded, and one extra minute of centrifugation at 10,000 g was performed to dry the column. Finally, the column was placed into a new 2 ml tube for final elution with 50 μl of Solution C6 and centrifugation at 10,000 g during 30s. For DNA quality control, two serial dilutions of the DNA samples were used. Genomic DNA was quantified using SYBRGreen I (Sigma–Aldrich, Merck) and the total bacterial load in the DNA sample was estimated by a real-time PCR assay with primers described in Nadkarni et al., 2002 (Nadkarni et al., 2002) (forward 5’-TCCTACGGGAGGCAGCAGT-3’ and reverse primer 5’-GGACTACCAGGGTATCTAATCCTGTT-3’), using a 7900 HT Fast Real-Time PCR System (Applied Biosystems).

For library preparation, the DNA samples were normalized according to their bacterial DNA content to be used as a template to prepare 16S rRNA libraries (region V3–V4). The 16S rRNA V3–V4 region was amplified with primers previously described (Willis et al., 2018), but the library preparation protocol was slightly modified. First, normalized DNA samples were used to amplify the V3–V4 regions of the 16S ribosomal RNA gene, in a limited cycle PCR. The PCR was performed in a 25 μl volume with 0.08 μM primer concentration and NEBNext Q5 Hot Start HiFi PCR Master Mix (ref. M0543L, New England Biolabs). The cycling conditions were an initial denaturation of 30 s at 98°C followed by 5 cycles of 98°C for 10 s, 55°C for 5 min, and 65°C for 45 s. After this first PCR, a second PCR was performed in a total volume of 50 μl. The reactions comprised NEBNext Q5 Hot Start HiFi PCR Master Mix and Nextera XT v2 adaptor primers. PCR was carried out to add full-length Nextera adapters: initial denaturation of 30 s at 98°C followed by 17 cycles of 98°C for 10 s, 55°C for 30 s, and 65°C for 45 s, ending with a final elongation step of 5 min at 65°C. Libraries were purified using AgenCourt AMPure XP beads (ref. A63882, Beckman Coulter) with a 0.9X ratio according to the manufacturer’s instructions and were analyzed using Fragment Analyzer (ref. DNF-915, Agilent Biosystems) to estimate the quantity and check size distribution. A pool of normalized libraries was prepared for subsequent sequencing. Final pools were quantified by qPCR using the Kapa library quantification kit for Illumina Platforms (Kapa Biosystems) on an ABI 7900HT real-time cycler (Applied Biosystems). Sequencing was performed on an Illumina MiSeq with 2 × 300 bp reads using v3 chemistry with a loading concentration of 18 pM. To increase the diversity of the sequenced, 10% of PhIX control libraries were spiked in.

Negative controls of the PCR amplification steps were routinely performed in parallel using the same conditions and reagents. Our control samples systematically provided no visible band or quantifiable DNA amounts. The ZymoBIOMICS™ Microbial Community DNA Standard (ref. D6306, Zymo) was amplified and sequenced in the same manner as all other experimental samples.

### 2.3 Bioinformatics and statistical analysis

Raw data were processed by using the Dada2 pipeline (v. 1.12.1) (Callahan et al., 2015). Low-quality reads were filtered and trimmed out based on the observed quality profiles by using the *filterAndTrim* function, truncating forward and reverse reads below 290 and 230, respectively, and considering a value of 2 as the maximum expected error. Furthermore, 10 reads from the start of each read were removed. We combined identical sequencing reads into unique sequences, made a sample inference from a matrix of estimated learning errors and merged paired reads. Chimeric sequences were removed by using the *removeBimeraDenovo* function, and taxonomy was assigned utilizing the SILVA 16S rRNA database (v.138) (Quast et al., 2013).

Two negative controls from DNA extraction were analyzed to assess possible sources of contamination and removed for further analysis. The resulting Amplicon Sequence Variant (ASV) table was merged with the metadata creating a *phyloseq* (v. 1.26.1) (McMurdie and Holmes, 2013) object. We filtered out taxa with fewer than 100 reads and with a relative abundance less than 0.1% or present in less than 5% of the samples.

Statistical analyses were performed using the 4.1.2 R version. In order to adjust for differences in the number of reads across samples and allow a proper alpha diversity comparison (Willis, 2019), the data were sampled at a value of 42,321 (rarefaction efficiency index = 0.99 (Hong et al., 2022), the minimum number of reads (**Supplementary Figure S2**).

To assess the alpha diversity of the samples four indexes were calculated (Chao, Simpson, Inverse Simpson and Shannon). However, since analogous results were obtained, only the Shannon index is reported in this study, which considers the differences in the abundance of each species and is the most commonly used diversity metric (Reese and Dunn, 2018). In addition, the mean and range richness of the samples at all taxonomical levels were plotted for each time point. Furthermore, the OTUs found in immediately frozen samples and not found anymore were listed.

**Figure 2:**
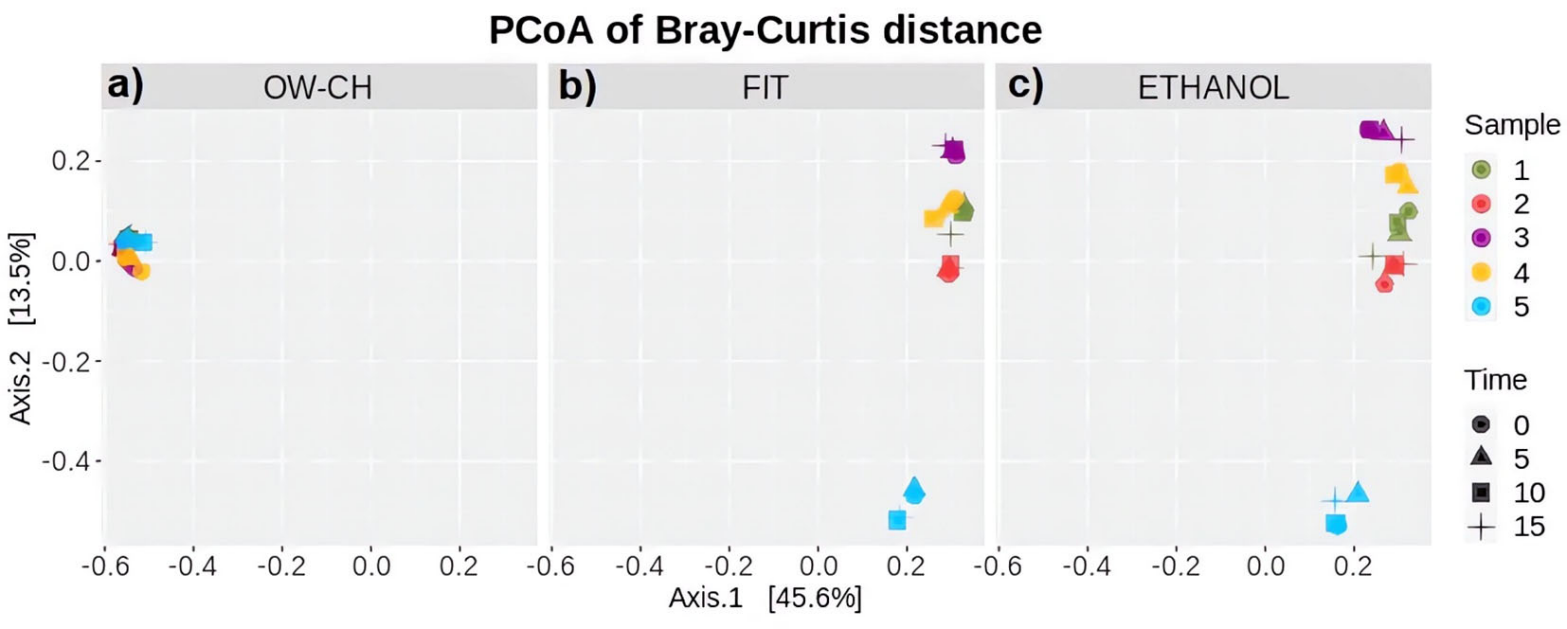
Principal Coordinates Analysis based on Bray–Curtis dissimilarity, illustrating beta diversity of microbial communities. The analysis is stratified by collection method: **(a)** OW-CH; **(b)** FIT; and **(c)** ETHANOL. Each point represents a sample, with colors indicating different donors and shapes representing the time elapsed at room temperature before freezing (0, 5, 10, and 15 days).

For the purpose of studying beta diversity, Bray–Curtis, Jaccard, unweighted UniFrac and weighted UniFrac dissimilarity distances were considered (Plantinga and Wu, 2021; By IMPACTT investigators, 2022), but since similar results were obtained, only the Bray–Curtis dissimilarity is reported. The projections of the individuals were plotted in one of the three plots depending on the collection method (**Figure 2**). Furthermore, the shapes were plotted according to the days staying at room temperature and colored according to the sample number.

Since the sample size of this stability study was small, the statistical analysis was focused on the estimation of changes and their 95% confidence intervals. Linear mixed models (LMM) were used to estimate the change in alpha diversity over the time points 0, 5, 10 and 15 days. LMMs account for the correlations between data including the subject as a random effect. Estimated marginal means (EMMs) were used to estimate differences among time points.

Multiple analysis of variance (MANOVA) was conducted to compare the abundance of the top 5 phyla and the top 20 orders with the number of days that the sample remained at room temperature before being frozen.

A sensitivity analysis was performed, removing one subject that showed a pattern considerably different from others.

The dataset that was generated and analyzed in our study is available at the Zenodo repository (DOI: 10.5281/zenodo.7684999, accessed on 28th February 2023).

## 3 Results

### 3.1 Comparing alpha and beta diversity between methods

**Figure 3:**
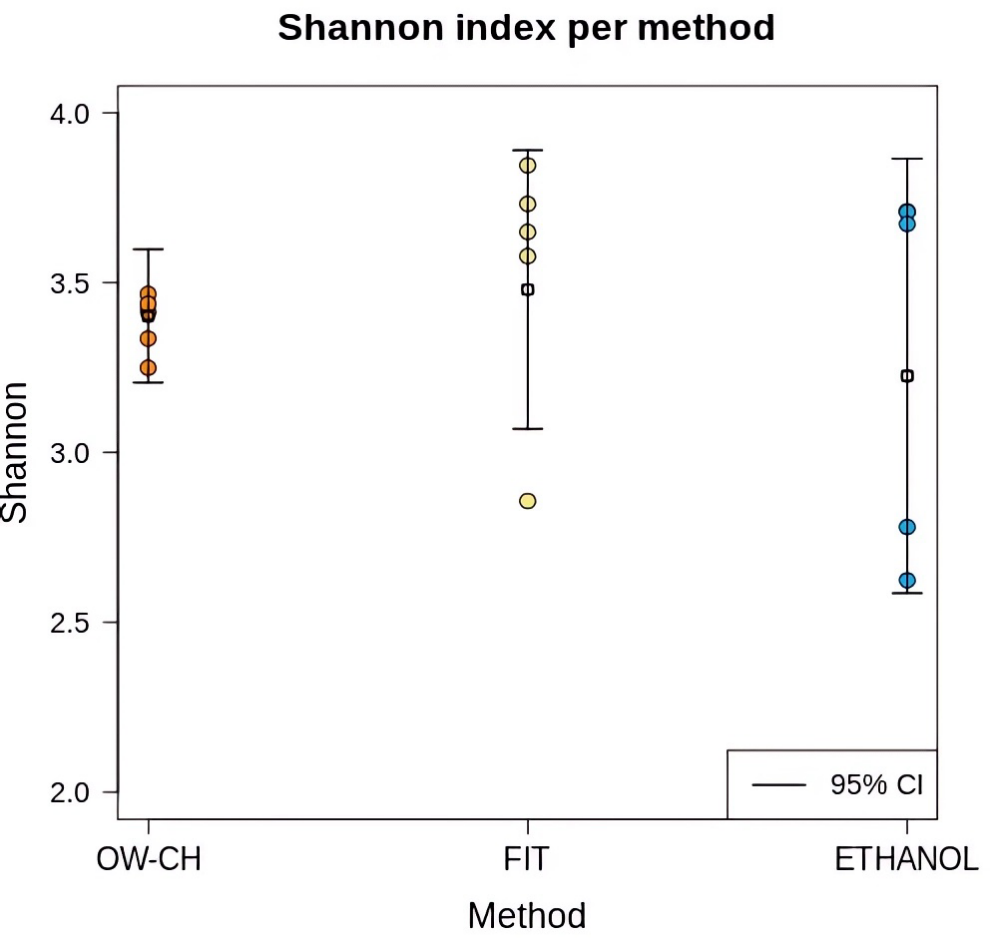
Shannon diversity index for each collection method in samples that were immediately frozen. Individual sample values are displayed as dots, with mean values and 95% confidence intervals represented by horizontal line.

At time 0, the Shannon diversity index showed only minor differences between the 70% ethanol and FIT stool collection methods (difference = 0.23, 95% CI: 0.18–0.65). However, samples preserved in 70% ethanol exhibited greater dispersion in diversity values. The alpha diversity of stool samples, as measured by the Shannon index, was comparable to that of oral wash (OW-CH), despite the distinct microbial composition observed in these samples (**Figure 3**).

**Figure 4:**
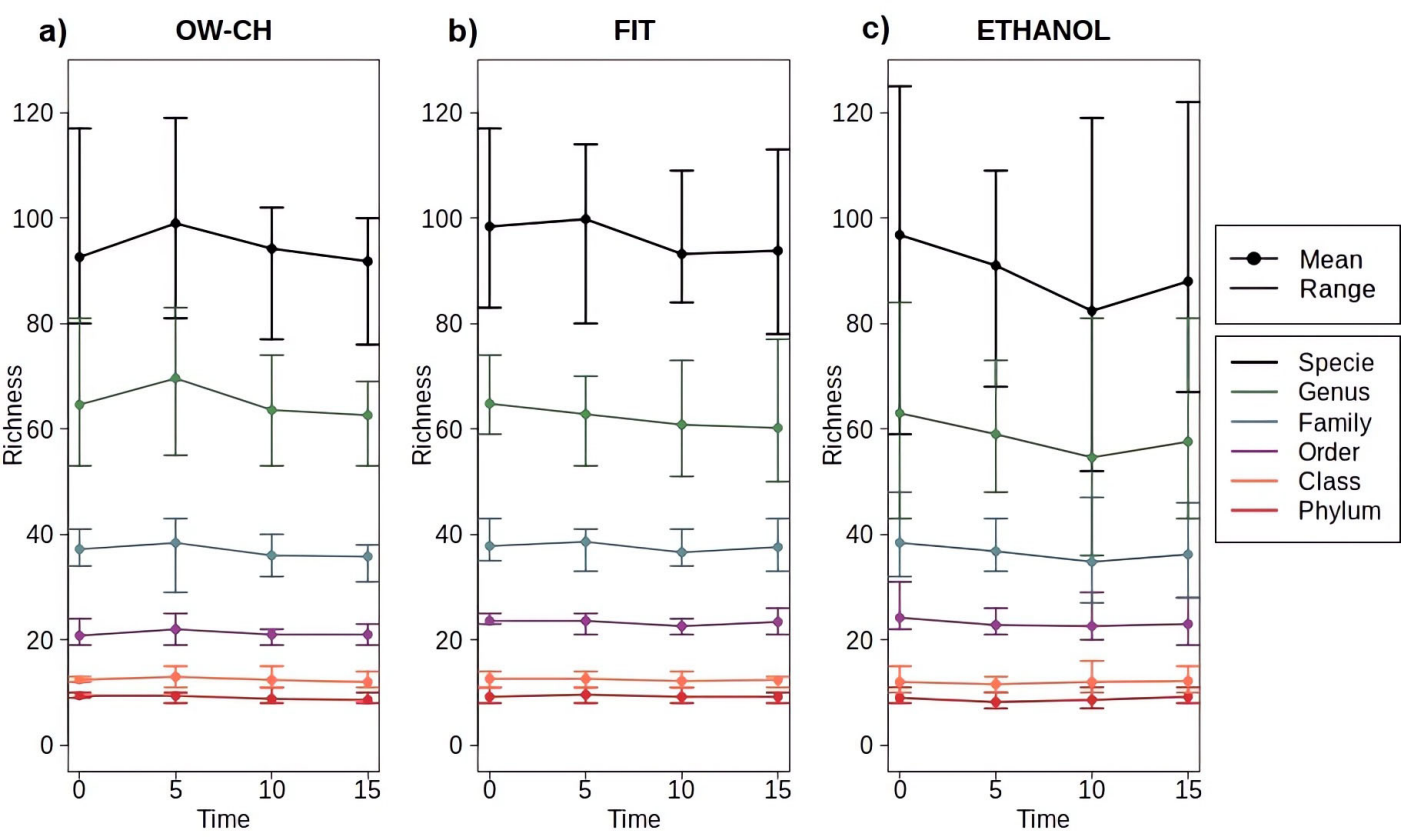
Mean and range of microbial richness across all taxonomic levels (from phylum to species) for samples stored at varying time points at room temperature before freezing, stratified by collection method: **(a)** OW-CH; **(b)** FIT; **(c)** ETHANOL.

Regarding OTU richness, no significant decrease, trend, or pattern was observed over time at room temperature (**Figure 4**). Across all taxonomic levels, richness remained largely stable, as indicated by overlapping confidence intervals and horizontal trends in the data. However, variation was only slightly greater at higher taxonomic levels, particularly in ethanol-preserved samples, which exhibited somewhat wider confidence intervals. Additionally, while overall taxonomic richness appeared comparable across methods, some OTUs detected in immediately frozen samples were absent in those stored at room temperature (**Supplementary Table S3**). Nevertheless, no single OTU was consistently lost across all three methods when immediate freezing was not applied.

**Figure 5:**
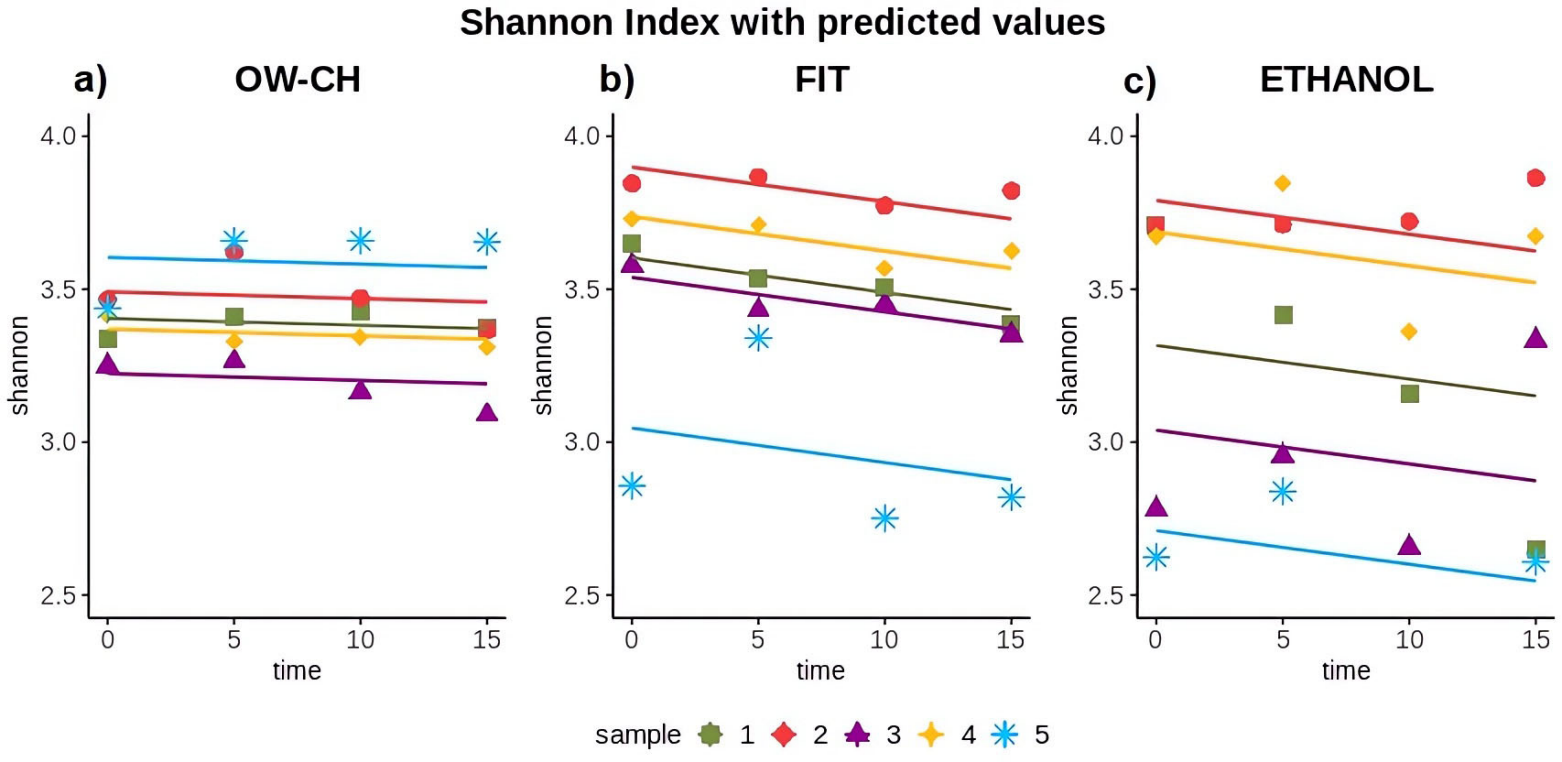
Shannon diversity index over time for each collection method: **(a)** OW-CH; **(b)** FIT; **(c)** ETHANOL.. Predicted values from Generalized Linear Mixed Models are included to assess diversity trends over time.

Regarding beta diversity, the distribution of the oral microbiota (**Figure 2a**) is clearly distinct from that of the stool microbiota (**Figures 2b, c**). However, both preservation methods for stool samples show strong concordance when assessed using Bray–Curtis dissimilarity distance. Additionally, individual projections appear grouped by subject rather than by time, indicating that storage duration at room temperature did not introduce noticeable patterns or shifts in microbial composition. Notably, subject 5 was more distant from the others along the second axis in stool samples, a finding that aligns with this subject’s lower alpha diversity (**Figure 5**).

### 3.2 Stability of the samples

The Shannon index for oral samples preserved in chlorhexidine remained stable over the 15 days at RT, showing no substantial trend and a 5-day mean decrease of 0.32% (**Table 1**; **Figure 5**). However, the alpha diversity index decreased over time for stool samples, with a 5-day mean decrease of 1.58% for FIT and 1.67% for ethanol preservation.

**Table 1:** Slopes of the generalized linear mixed models for Shannon index with 95% confidence intervals and 5-day percentage mean decrease.

Method	Shannon Index			
	Average time 0	Slope coefficient	95% CI	5-day % decrease
OW-CH	3.38	−0.002	(−0.009; 0.005)	−0.32
FIT	3.53	−0.011	(−0.021; −0.001)	−1.58
ETHANOL	3.30	−0.011	(−0.034; 0.012)	−1.67

Nevertheless, although the shipment of the samples is not expected to take so long, the Shannon Index variation over the 15 days was only −3.68% for the FIT collection method (**Table 2**). For the 70% ethanol and oral wash collection methods, the shifts were −2.12% and −0.59%, respectively. The pairwise comparison of time points 0 and 5 did not show a major change.

**Table 2:** Mean at time 0 (standard error) and absolute difference and percentage of change of pairwise comparisons of the time points with respect to day 0.

Shannon Index						
	OW-CH		FIT		ETHANOL	
Mean time 0 (s.e)	3.38 (0.07)		3.53 (0.16)		3.30 (0.24)	
Time point	Absolute difference	% Change	Absolute difference	% Change	Absolute difference	% Change
0–5	−0.07	2.37	−0.04	1.42	−0.05	1.52
0–10	−0.03	0.89	0.12	−3.40	0.27	−8.48
0–15	0.02	−0.59	0.13	−3.68	0.07	−2.12

Average values were derived from the estimated marginal means of the LMM model.

While some inter-individual differences were observed, particularly for subject 5, who exhibited lower alpha diversity values and a slightly more pronounced decline over time (**Figure 5**), these variations did not substantially alter the overall trend. This suggests that, despite minor fluctuations, microbial diversity remains largely stable under the tested preservation conditions

Additionally, a sensitivity analysis excluding subject 5—who was an outlier in the Principal Coordinates Analysis (PCoA) plot using both Jaccard and Bray–Curtis dissimilarity matrices—confirmed that the results remained consistent.

### 3.3 Top 5 phyla stability over time

The results from the MANOVA (**Supplementary Table S4**) indicated no significant differences in the relative abundances of the most common phyla (*Firmicutes*, *Bacteroidota*, *Actinobacteriota*, *Proteobacteria* and *Campylobacterota*) over time at room temperature. This suggests that the preservation methods maintained the overall microbial composition with minimal fluctuations.

Among the phyla analyzed, those with the highest percentage changes over 5 days were all minority phyla (<3.5% relative abundance), including *Campylobacterota* in OW-CH (13.26%), *Desulfobacterota* in FIT (24.60%), and *Fusobacteriota* in ethanol (−19.7%). However, the confidence intervals for these variations were wide, indicating potential variability across samples rather than a consistent trend.

Notably, the most abundant phyla—*Firmicutes* and *Bacteroidota*—remained relatively stable across all preservation methods. The largest shift observed among dominant phyla was a 12.18% increase in *Actinobacteriota* in ethanol, but with a broad confidence interval (−3.14 to 27.50), suggesting variability rather than a systematic effect.

**Figure 6:**
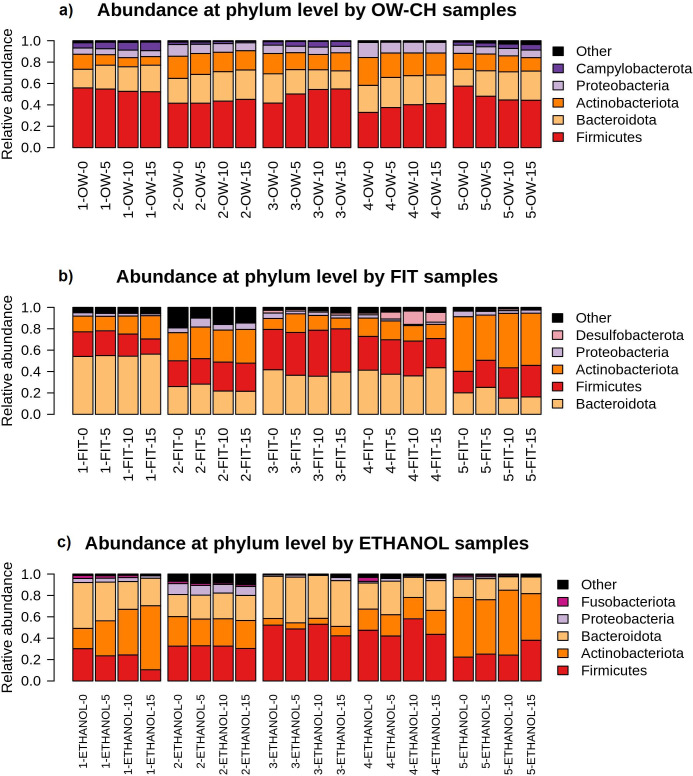
Relative abundance of the main bacterial phyla detected in each sample, stratified by collection method (**(a)** OW-CH; **(b)** FIT; **(c)** ETHANOL) and individual donor. Changes in phylum-level composition across different time points at room temperature are shown.

The bar plots in **Figure 6** further illustrate this stability, showing consistent relative abundances of the major phyla across all time points for each method. While some fluctuations can be observed, particularly in the less abundant phyla, these variations do not appear to affect the overall microbial composition in a meaningful way.

### 3.4 Top 20 orders stability over time

A MANOVA analysis assessed microbial stability over time, showing that most of the top 20 bacterial orders remained relatively stable, with only a few declining noticeably (**Supplementary Table S5**).

Over 5 days, in OW-CH, most frequent orders remained stable, but *Actinomycetales* declined (−10.75%), while some low-abundance orders saw more pronounced reductions: *Burkholderiales* (−16.73%), *Bifidobacteriales* (−17.21%), *Synergistales* (−25.03%), and *Spirochaetales* (−42.37%). In FIT samples, frequent orders showed little change, with the exception of *Erysipelotrichales* (−16.71%). For ethanol samples, most frequent orders were stable, though *Fusobacteriales* declined (−19.76%), along with the less frequent Veillonellales-*Selenomonadales* (−28.90%).

**Figure 7:**
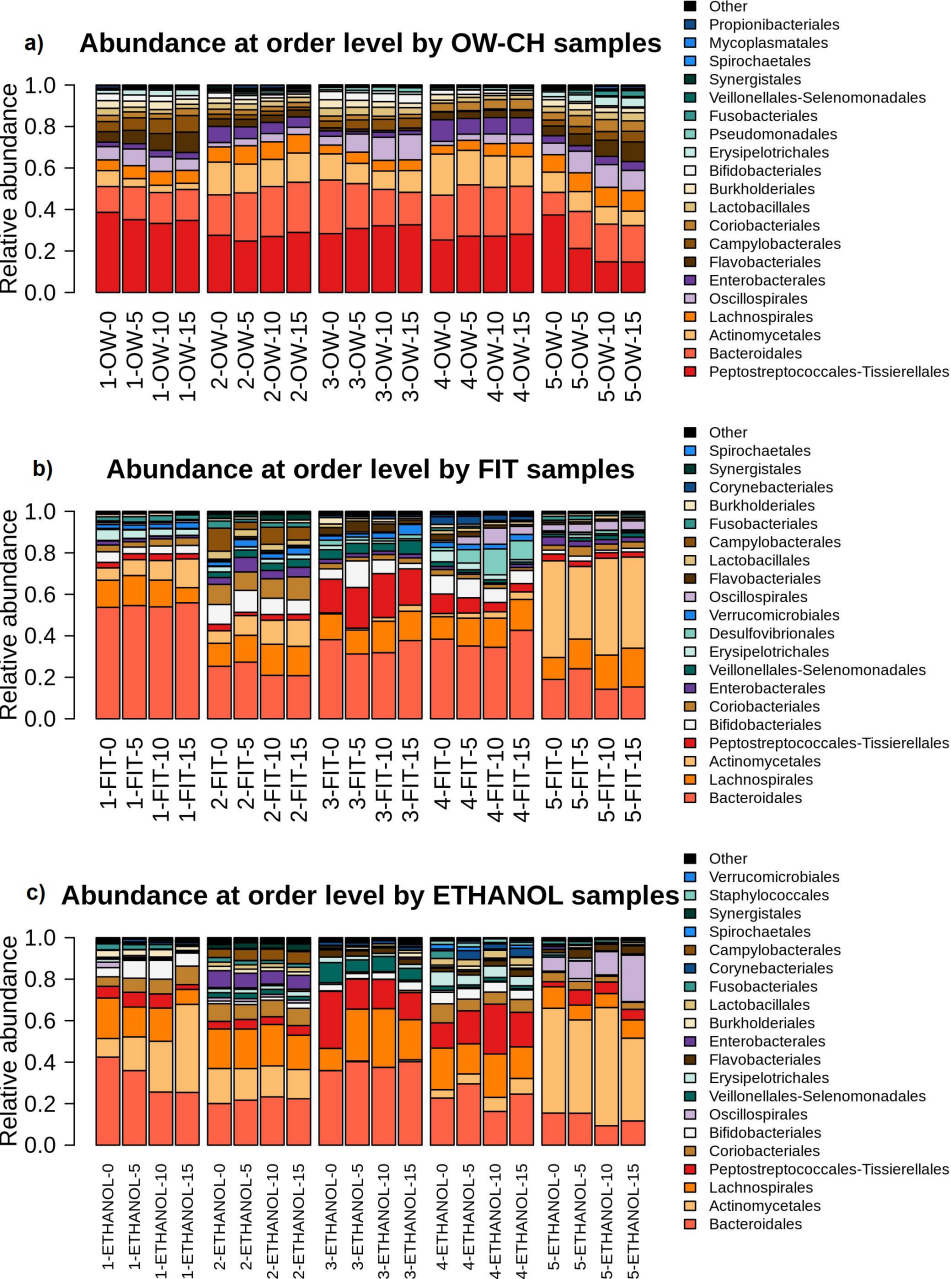
Relative abundance of the main bacterial orders detected in each sample, stratified by collection method (**(a)** OW-CH; **(b)** FIT; **(c)** ETHANOL) and individual donor. Temporal shifts in microbial composition at the order level are visualized.

Although sample 5 from the fecal microbiome showed a distinct order-level profile compared to other samples. Despite this, it followed similar stability patterns across preservation methods, as illustrated in **Figure 7**.

## 4 Discussion

The stability of microbiome samples at room temperature for 15 days was investigated for oral wash samples preserved in chlorohexidine and two fecal collection methods (FIT and 70% ethanol). We found that oral microbiome diversity and composition were, in general, very stable during the 15 days at room temperature. For both fecal preservation methods, however, a small decrease in diversity was observed, mainly after day 5, with the samples stored in ethanol showing more heterogeneity. Between subjects, variability was of similar magnitude to the fluctuations in alpha diversity observed over time.

Although the microbial profile stability has previously been validated for 95% ethanol (Song et al., 2016; Byrd et al., 2019; Marotz et al., 2021), several studies caution against the use of 70% ethanol since it is found to be less stable than other collection methods stored for 4 days (Sinha et al., 2016; Byrd et al., 2019), 1 week (Sinha et al., 2016; Marotz et al., 2021) and 8 weeks (Song et al., 2016). Other works do not report significant changes between immediately frozen samples and the 70% ethanol microbiome samples stored for 8 weeks at room temperature (Park et al., 2020). Our results are in agreement with previous works that reported no significant changes between immediately frozen samples and 70% ethanol samples for microbiome studies, at least for 5 days at room temperature, which is the usual shipment time.

Regarding the FIT collection method, several studies recommend its use in epidemiological studies. It has been proved that, in terms of alpha diversity, FIT samples remain stable for one week at RT (Gudra et al., 2017; Byrd et al., 2019; Krigul et al., 2021). Our work agrees with previous research, not showing significant changes in the composition of the samples.

Previous studies used Scope^®^ oral wash to preserve oral microbiome samples, and its stability at room temperature was already verified (Vogtmann et al., 2019; Yano et al., 2020; Wu et al., 2021); however, it is not easily found in Europe. As chlorhexidine has been commonly used in many clinical trials where effective results have been proven in reducing the proliferation of bacterial (Eick et al., 2011; James et al., 2017; Brookes et al., 2020; Xiang et al., 2021), we opted for Lacer^®^ Chlorhexidine oral wash to preserve the samples. To the best of our knowledge, the stability of Lacer^®^ oral wash samples at room temperature has not been previously studied. Our results sustain that the alpha diversity of the samples remained stable for 15 days at RT with no major shifts.

The choice of the sample collection method in a microbiome study plays a crucial role in obtaining reliable results (Wu et al., 2021). The mechanisms affecting bacterial 16S rRNA gene abundance and reduction in each storage group may vary depending on the preservative used. FIT samples contain a stabilizing buffer that prevents bacterial growth and preserves DNA integrity, which may contribute to their observed stability over time (Krigul et al., 2021). However, FIT was originally designed for detecting blood in feces and not specifically for microbiome studies, which may introduce bias in microbial composition (Vandeputte et al., 2017). On the other hand, 70% ethanol is commonly used as a fixative due to its ability to inactivate microbes and prevent DNA degradation. Nevertheless, some studies have suggested that it may not preserve microbial diversity as effectively as other methods (Sinha et al., 2016). Our results show that, while ethanol-preserved samples remained relatively stable for up to 5 days, variations in specific taxa were observed, particularly in certain individuals, which aligns with previous reports (Park et al., 2020). Finally, chlorhexidine-based mouthwashes provide an antimicrobial effect that helps maintain DNA integrity, making them a suitable option for oral microbiome preservation (Eick et al., 2011). This may explain why we observed minimal changes in oral samples stored in chlorhexidine for 15 days. Researchers should carefully consider these advantages and limitations when selecting a method, aligning it with the study’s objectives and the nature of the samples being investigated.

Although we are aware that the small sample size of the present study is not powered to perform statistical tests, the estimates of change and 95% confidence intervals allow a reasonable assessment of the quality of the sample preservation methods. On average, the magnitude of the changes in alpha diversity was smaller than 2%, allowing a reasonable assessment of the quality of the sample preservation methods. Phylum compositions showed good temporal stability, except for fecal samples preserved in ethanol in subject number 5, which had a different microbiome pattern. Furthermore, a shift was observed in individual 1 for the 70% ethanol samples, while *Actinobacteriota* increased and *Firmicutes* and *Bacteroidota* decreased. Park et al. (2020) also reported significant differences in the relative abundance of the two latter phyla compared to control samples. Regarding order compositions, although slight relative abundance differences could be found in a few of the low-abundance orders, the main ones remained stable during the time of the study.

## 5 Conclusion

To conclude, the stability of the samples regarding diversity and composition was verified for the chlorohexidine oral wash and two fecal methods (FIT and 70% ethanol). Alpha diversity was maintained over 15 days at room temperature for the chlorohexidine oral wash. For fecal samples, both 70% ethanol and FIT showed a decrease in diversity over time but a small decrease during the first 5 days. No substantial changes in overall abundance were observed, with minor differences noted in some less abundant phyla and orders.

## Data Availability

The datasets presented in this study can be found in online repositories. The names of the repository/repositories and accession number(s) can be found below: https://www.ebi.ac.uk/ena, PRJEB67775; https://zenodo.org/records/7684999, 7684999.
